# COVID-19 patients display changes in lymphocyte subsets with a higher frequency of dysfunctional CD8lo T cells associated with disease severity

**DOI:** 10.3389/fimmu.2023.1223730

**Published:** 2023-09-21

**Authors:** Luisina Ines Onofrio, Constanza Marin, Jeremías Dutto, María Belén Brugo, Ruth Eliana Baigorri, Sabrina Noemi Bossio, Juan Nahuel Quiróz, Laura Almada, Federico Ruiz Moreno, Carolina Olivera, Silene M. Silvera-Ruiz, Nicolás Eric Ponce, Paula Alejandra Icely, María Carolina Amezcua Vesely, Laura Fozzatti, María Cecilia Rodríguez-Galán, Cinthia Carolina Stempin, Laura Cervi, ImmunoCovid-CBA, Fabio Cerban, Belkys Angélica Maletto, Eva Virginia Acosta Rodríguez, Mariana Bertone, Claudio Daniel Abiega, Daiana Escudero, Adrián Kahn, Juan Pablo Caeiro, Mariana Maccioni, Claudia Cristina Motrán, Adriana Gruppi, Claudia Elena Sotomayor, Laura Silvina Chiapello, Carolina Lucia Montes

**Affiliations:** ^1^ Departamento de Bioquímica Clínica, Facultad de Ciencias Químicas, Universidad Nacional de Córdoba, Córdoba, Argentina; ^2^ Centro de Investigaciones en Bioquímica Clínica e Inmunología (CIBICI), Consejo Nacional de Investigaciones Científicas y Técnicas (CONICET), Córdoba, Argentina; ^3^ Instituto Universitario de Ciencias Biomédicas de Córdoba (IUCBC), Hospital Privado Universitario de Córdoba, Córdoba, Argentina

**Keywords:** COVID-19, SARS-CoV-2, cytotoxic CD4 + T cells, CD8 + T cells, T cells dysfunction

## Abstract

This work examines cellular immunity against SARS-CoV-2 in patients from Córdoba, Argentina, during two major waves characterized by different circulating viral variants and different social behavior. Using flow cytometry, we evaluated the main lymphocyte populations of peripheral blood from hospitalized patients with moderate and severe COVID-19 disease. Our results show disturbances in the cellular immune compartment, as previously reported in different cohorts worldwide. We observed an increased frequency of B cells and a significant decrease in the frequency of CD3^+^ T cells in COVID-19 patients compared to healthy donors (HD). We also found a reduction in Tregs, which was more pronounced in severe patients. During the first wave, the frequency of GZMB, CD107a, CD39, and PD-1-expressing conventional CD4^+^ T (T conv) cells was significantly higher in moderate and severe patients than in HD. During the second wave, only the GZMB^+^ T conv cells of moderate and severe patients increased significantly. In addition, these patients showed a decreased frequency in IL-2-producing T conv cells. Interestingly, we identified two subsets of circulating CD8^+^ T cells with low and high CD8 surface expression in both HD and COVID-19 patients. While the percentages of CD8^hi^ and CD8^lo^ T cells within the CD8^+^ population in HD are similar, a significant increase was observed in CD8^lo^ T cell frequency in COVID-19 patients. CD8^lo^ T cell populations from HD as well as from SARS-CoV-2 infected patients exhibited lower frequencies of the effector cytokine-producing cells, TNF, IL-2, and IFN-γ, than CD8^hi^ T cells. Interestingly, the frequency of CD8^lo^ T cells increased with disease severity, suggesting that this parameter could be a potential marker for disease progression. Indeed, the CD8^hi^/CD8^lo^ index helped to significantly improve the patient’s clinical stratification and disease outcome prediction. Our data support the addition of, at least, a CD8^hi^/CD8^lo^ index into the panel of biomarkers commonly used in clinical labs, since its determination may be a useful tool with impact on the therapeutic management of the patients.

## Introduction

Coronavirus disease 2019 (COVID-19), triggered by severe acute respiratory syndrome-coronavirus-2 (SARS-CoV-2), has had a profound impact on the world since its initial emergence in late 2019. In response to the pandemic, countries worldwide implemented diverse non-pharmaceutical intervention strategies. Argentina promptly implemented a comprehensive of public health and social measures against SARS-CoV-2 infection. The first confirmed case in Argentina was reported on March 3, 2020. In the absence of effective antiviral treatment or vaccines against the virus, the Argentine government decreed mandatory social isolation on March 19, 2020. There were several waves of SARS-CoV-2 infection from 2020 to 2022, with the first two peaking in October 2020 and May 2021 (1). During the first wave, the prevalent strains in circulation were B.1.499 and N.3 lineages (2), while in the second wave, the gamma, lambda, and alpha variants of SARS-CoV-2 emerged as the dominant strains, followed by non-Variants of Concern (VOC) non Variants of Interest (VOI) (3).

The range of clinical manifestations associated with COVID-19 varies from asymptomatic or symptomatic forms to severe illness. Countless studies have been conducted due to the global dissemination and devastating consequences of COVID-19 on individuals, focusing on risk factors, the progression of the disease, and the immune response mediated by different cellular and molecular components. Concerning immunity, hosts develop different anti-SARS-CoV-2 strategies in response to infection. The level of the initial viral load (4) and the effectiveness of the innate immunity, mainly that triggered by type I interferons (5, 6), seem to be key in establishing a foundation for both the adaptive response and the clinical course of COVID-19. It has been reported that adaptive immunity plays a critical role in controlling SARS-CoV-2 infection and is the major determinant of the evolution of the infection (7, 8). Severe disease, dominated by pneumonitis (9–11), is associated with a gradual reduction in viral load, early and inflammation with elevated inflammatory cytokines (11) and impaired T cell response (12) associated with activation and lymphopenia (13). Effective control of the virus is associated with a Th1-type response, whereas patients with severe disease exhibit a Th2- type response (14).

As for other viral infections, early cytotoxic CD8^+^ T cell responses correlate with efficient viral clearance (14). In acute COVID-19, high levels of expression of cytolytic effector molecules by CD8^+^ T cells are associated with improved clinical outcomes (15). Paradoxically, an excessive T cell activation appear to have a detrimental effect, as it correlates with unfavorable clinical outcomes (16). Similarly, the expression of activation markers linked to exhaustion, such as PD-1 and Tim-3, are associated with bad disease progression (13). Interestingly, by using single-cell transcriptomic analysis conducted four weeks after infection, Bieberich et al. (17) reported a significant expansion of cytotoxic populations in both CD8+ T cells and CD4+ T cells. However, it is worth noting that cytotoxic CD4+ subsets are not prominently featured in the memory response. Compared with those non-hospitalized, hospitalized patients have higher proportions of cytotoxic follicular helper and cytotoxic CD4+ T cells that respond to SARS-CoV-2. Additionally, there is a decreased proportion of SARS-CoV-2-reactive regulatory T cells (Treg) in these hospitalized patients (18). The importance of Treg in the disease course remains uncertain, but their reduction would explain the high degree of inflammatory response observed in patients. As in many reports (reviewed in (19), we have described elevated levels of inflammatory cytokines in COVID-19 patients, with differential intensity in the signatures of systemic cytokines and chemokines measured in the serum of patients recruited during the first and the second waves of SARS-CoV-2. Additionally, we observed that concurrent pre-existing comorbidities do not significantly contribute to the higher levels of systemic cytokines and chemokines in these patients. In addition, we detected that patients recruited during the second wave of infection were characterized by a younger age and a lower prevalence of concurrent comorbidities compared to those from the first outbreak (20).

Regarding the B cells compartment, COVID-19 patients exhibited changes in different B cell subsets (21). The infection triggers a polyclonal B cell response with limited clonal expansion (22). During hospitalization, some clones were identified as plasmablasts and these persisted for up to 1 year as memory B cells (22).

In summary, the extensive perturbations in the immune system involving both innate and adaptive leukocytes driving an unbalanced response in severe COVID-19 patients justified a deep investigation.

Most of the information on cellular immunity that we previously mentioned comes from studies conducted in China, Europe, and the United States of America. In the current work, we studied cellular immune response against SARS-CoV-2 infection in patients from Córdoba, Argentina. The Argentine population is a mixture of three ancestral populations: European (mostly of Spanish and Italian descent), Native American, and Sub-Saharan African, with differences in terms of demographics and dietary patterns compared to Oriental and Mediterranean regions (23, 24). Considering that HLA-mediated immunity in COVID-19 is relevant (25) and that the type of diet may condition immunity (26, 27), the present study aimed to investigate T cell immune response to SARS-CoV-2 infection in an Argentine cohort. Patients hospitalized in Córdoba with moderate and severe COVID-19 were studied during the first and second waves of infection.

Our data provide crucial insight not only at a local level, delineating the T cell response of COVID-19 patients in Córdoba, but also at the international level, confirming data already reported, and describing for the first time that an increased frequency of dysfunctional CD8^lo^ T cells is associated with severity in COVID-19 patients.

## Methods

### Ethics statement

This study received approval from the “Registro Provincial de Investigación en Salud (RePIS)” (Provincial Registry of Health Research), Córdoba, Argentina under number 4039. Additionally, it was also approved by the Institutional Review Board (IRB) of the Hospital Privado Universitario de Córdoba (HPUC), Córdoba, Argentina. Prior to their participation, all enrolled patients provided written informed consent in accordance with the Declaration of Helsinki, and their data were protected as per Argentine law N° 25.326. Patient recruitment took place during the first and second waves of the SARS-CoV-2 pandemic in Córdoba, Argentina, between October-December 2020 and February-June 2021, respectively (1, 28) (https://www.argentina.gob.ar/salud/coronavirus-COVID-19. The B.1.499 and N.3 lineages were the most prevalent circulating strains during the first wave (2), while the gamma, lambda, and alpha SARS-CoV-2 variants were predominant during the second wave followed by non-VOC non-VOI variants (3).

### Participants

Diagnosis of SARS-CoV-2 infection was performed using a nucleic acid amplification test for SARS-CoV-2, in accordance with the guidelines provided by the Argentine Health Ministry. Reverse transcriptase polymerase chain reaction (RT-PCR) (PerkinElmer^®^, Massachusetts, U.S) analysis was conducted on samples obtained from nasal and pharyngeal swabs (1). Only hospitalized, primo-infection cohorts were recruited, and were classified as patients with moderate (n=52) or severe COVID-19 disease (n=32) according to clinical parameters (29, 30). In the current study, we employed multiple risk assessment scores to quantify the severity of COVID-19 disease (29, 30).. Additionally, we integrated clinical judgement as a crucial determinant in the process of assigning appropriate admission as patients with moderate or severe disease. The categorization into moderate or severe was determined upon admission. The current study did not encompass the assessment of individuals initially classified as patients with moderate disease (MD) who subsequently worsened and progressed to severe disease (SD). All patients recruited during either the first or second wave of the pandemic did not experience symptoms compatible with COVID-19 before recruitment or had a previously positive SARS-CoV-2 nucleic acid amplification test. The cohort of patients did not receive any immunomodulatory medication or specific anti-inflammatory therapies previous hospitalization and at the time of sample obtention.

Samples from SD and MD patients were collected between days 0-4 after hospital admission. The sampling time was comparable among patients. The hospitalization period exhibited variability among patients. Those with moderate disease were admitted for a span of 7 to 15 days, whereas individuals with severe disease required ICU stays lasting from 14 to 23 days.

Data related to age, gender, severity, and global mortality were collected in a database and are summarized in **Supplementary Table 1**. In the current study, we excluded patients with other active infections and patients with cancer, immunodeficiency, or terminal chronic diseases. Patients with pre-existent comorbidities such as diabetes, arterial hypertension, asthma, overweight, heart disease, dyslipidemia, hypothyroidism, and obesity were included. The demographic features, laboratory, clinical characteristics, and cytokine and chemokine serum concentrations of hospitalized COVID-19 patients were reported in an our previous report using the same cohort of patients (20)

A group of 24 healthy volunteers (aged 30- 60 years), were selected and recruited to participate in the study as healthy donors (HD). The cohort comprised 11 men and 12 women, reflecting a diverse representation of both genders.

HD had no prior history of SARS-CoV-2 infection, as determined by the absence of a positive SARS-CoV-2 nucleic acid amplification test, absence of compatible symptoms, and no history of close contact with confirmed COVID-19 cases based on epidemiological criteria (1, 29). Furthermore, the HD selected for this study had no history of chronic diseases and did not exhibit any symptoms suggestive of an infectious illness in the month leading up to sample collection. It is important to note that none of the participants, including the HD group, had received a COVID-19 vaccine prior to their inclusion in this study.

### Samples

Peripheral blood from HD and from COVID-19 patients on hospitalization was drawn by venipuncture into BD Vacutainer^®^ EDTA tubes BD. Peripheral blood mononuclear cells (PBMCs) were isolated from whole blood samples using Ficoll-Paque™ PLUS (GE Healthcare Bio-Science AB). Isolated PBMCs were cryopreserved in heat-inactivated fetal bovine serum (FBS; Natocor) containing 10% DMSO (Sigma-Aldrich) and stored in liquid nitrogen until use. Routine blood tests (neutrophil, lymphocyte and platelet counts, and serum CRP concentration) were conducted at the medical laboratory of the Hospital Privado Universitario de Córdoba. Patients with moderate and severe COVID-19 from both first and second waves presented marked lymphopenia in comparison to HD. HD: 2507 ± 625 cells/ul (1760-3950); MD: 1194± 698 cells/ul (253-3234); SD 1239± 969 cells/ul (50-3516).

### Flow cytometry

For surface phenotype studies, PBMCs were stained with a mixture of fluorochrome-labeled monoclonal antibodies (mAb) against the corresponding antigen, following procedures previously described (31). Fluorochromes and clones are detailed in **Supplementary Table S2**.

For intracellular staining, cells were fixed/permeabilized with Foxp3 Staining Buffer and Perm Wash Buffer (eBiosciences) following manufacturer’s indications. For *ex vivo* intracellular cytokines, we stimulated cells with 50 ng/ml PMA (SIGMA) and 1 µg/ml Ionomycin (SIGMA) cultured with Brefeldin A plus Monensin (eBioscience) for 4 h at 37°C. The CD107a staining, was performed stimulating cells as indicated before for 4 h in the presence of anti-CD107a mAb. In all cases, dead cells were excluded using LIVE/DEAD™ Fixable Aqua for 405nm (Invitrogen™).

The gating strategies used to recognize the cell subsets evaluated in the current study are shown in **Figure S1**. Samples were acquired in a BD LSR Fortessa flow cytometer (BD Biosciences) and data analyzed with FlowJo software version 10 (BD Bioscience).

### Statistical analysis

Statistical and graphical data analyses were performed with GraphPad Prism version 8.3 (GraphPad Software, San Diego, CA, USA) and R studio version R-4.0.2 (30). We conducted a linear discriminant analysis (LDA) using InfoStat statistical Software (Facultad de Ciencias Agropecuarias, UNC, Argentina). A k-nearest neighbor imputation based on Euclidean distance was used to handle missing data. *p*-values <0.05 were considered significant (two sides). The Shapiro-Wilk normality test was initially performed to determine the distribution of the datasets. Numerical variables were expressed as mean (± standard error of mean, SEM). The specific tests used are indicated in the legends for the figures.

## Results

### Analysis of the frequency of the main peripheral lymphocytes from COVID-19 patients in an Argentine cohort revealed alterations in the frequency of CD19^+^, CD3^+^, and Treg cells

**Figure 1:**
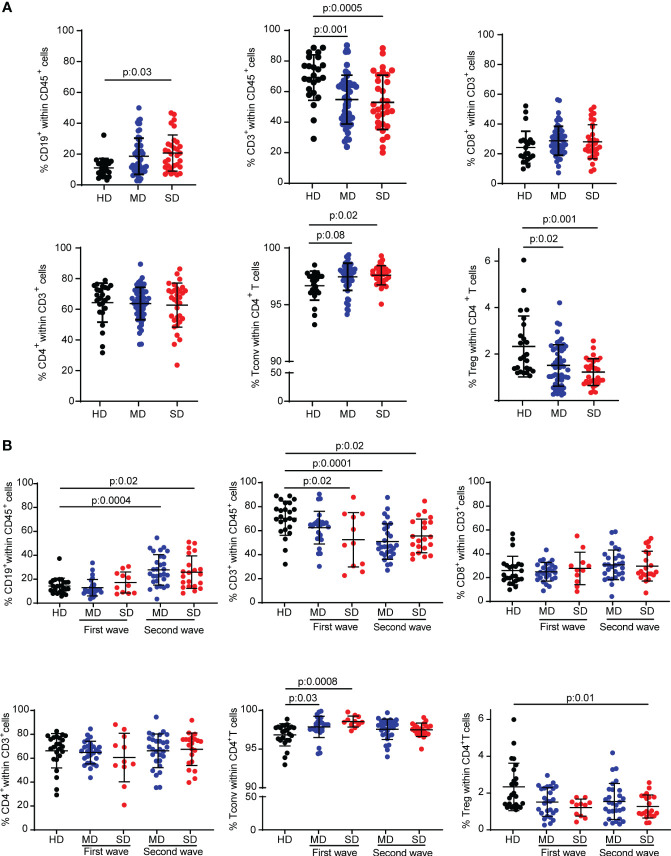
Frequency of different lymphocyte subsets from HD and COVID-19 patients of an Argentine cohort. Percentage of CD19^+^ cells, CD3^+^ cells, CD8^+^ T cells, total CD4^+^ T cells, Tconv (CD4^+^ Foxp3^-^) and Treg (CD4^+^ Foxp3^+^) T cells from PBMC samples from the entire cohort of hospitalized patients **(A)** or patients of the first and second wave analyzed separately **(B)**. Dots show individual measurements of groups: HD (black), MD COVID-19 patients (blue), and SD COVID-19 patients (red), and black lines show the mean frequency of each population +/- SEM. One-way analysis of variance (ANOVA) followed by the Tukey’s multiple comparisons test or the Kruskal-Wallis test followed by Dunn’s multiple comparisons test were used for statistical analysis when the measured variable had or had not met the normality assumptions, respectively. HD, healthy donors; MD, patients with moderate disease; SD, patients with severe disease.

In order to understand the impact of COVID-19 on cellular immunity of individuals residing in Argentina, we first evaluated the frequency of the main lymphocyte populations in peripheral blood from COVID-19 infected patients by flow cytometry. Using a gating strategy depicted in **Figure S1**, we analyzed the frequencies of total CD19^+^, CD3^+^, CD8^+^, CD4^+^, conventional CD4^+^ T (Tconv) (CD4^+^Foxp3^-^), and Treg (CD4^+^Foxp3^+^) lymphocytes from PBMCs of the entire cohort of hospitalized patients recruited during the first and second COVID-19 waves in Córdoba (Argentina) with moderate (MD) and severe (SD) COVID-19 disease. Recruited HD were used as controls. Interestingly, we observed that SD COVID-19 patients showed an increase in the frequency of B cells compared to HD (**Figure 1A**). Furthermore, MD and SD patients exhibited a significant decrease in the frequency of CD3^+^ T cells compared to HD. While the frequency of CD4^+^ and CD8^+^ T cells from COVID-19 patients was similar to that observed in HD, study of the CD4^+^ T cell compartment showed increased frequencies of Tconv in MD and SD patients concomitantly with a reduction of Treg populations, which was more pronounced in SD patients (**Figure 1A**).

Considering that the COVID-19 waves were characterized by the circulation of different SARS-CoV-2 strains or variants of concern (32) and by different average ages (**Supplementary Table S1**) and social behaviors (the confinement and quarantine measures were stricter during the first wave than during the second) of the population, we decided to analyze the immune cell populations according to the wave in which the samples were obtained. Stratifying the patients in this way, COVID-19 patients enrolled in the second wave, regardless of the severity of the disease, had a higher frequency of CD19^+^ B cells than HD. The T cell compartment exhibited a different behavior. Indeed, there was a reduction in the frequency of CD3^+^ T cells compared to HD in SD patients in both waves and in MD patients from the second wave. No significant differences were found in the frequency of CD8^+^ and CD4^+^ T cells from patients of each wave compared to HD. The reduction in Treg frequency was statistically significant in the population of SD COVID-19 patients from the second wave (**Figure 1B**).

### COVID-19 patients showed CD4^+^ T cells with an activation/cytotoxic phenotype in the first SARS-CoV-2 wave

**Figure 2:**
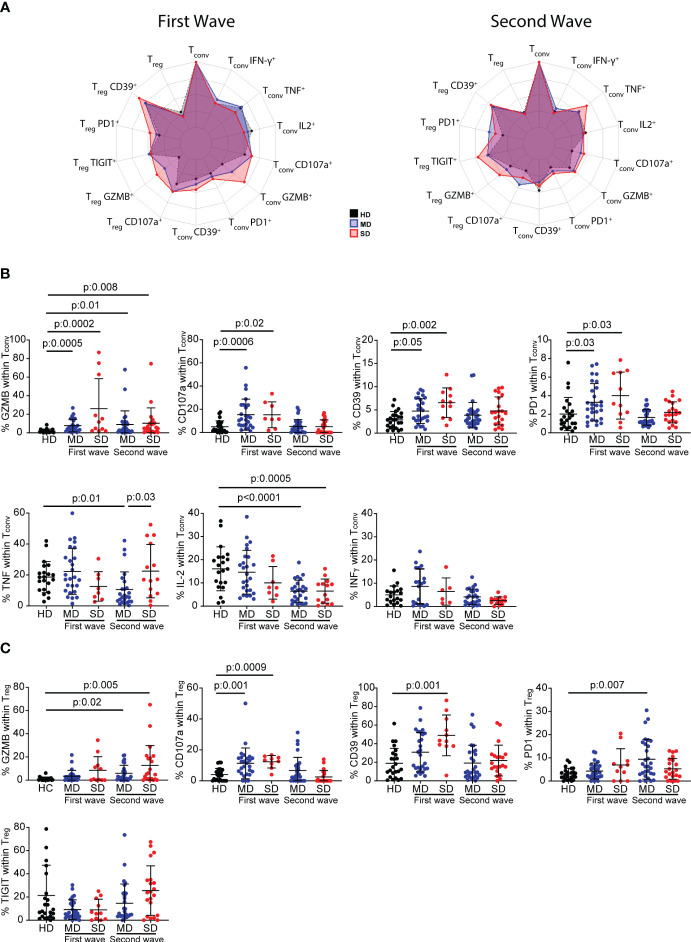
Frequency of inhibitory receptors and cytokine-producing CD4^+^ T cells from HD and COVID-19 patients. Radar plots depict the normalized average frequencies of Tconv (CD4^+^Foxp3^-^) and Treg (CD4^+^Foxp3^+^) expressing exhaustion, cytotoxic markers, and cytokines, from PBMCs of HD and SD and MD patients of the first and second waves **(A)**. Frequency of GZMB^+^, CD107a^+^, CD39^+^, PD-1^+^ and TNF^+^, IFN-γ and IL-2-producing Tconv cells, and GZMB^+^, CD107a^+^, CD39^+^, PD-1^+^ and TIGIT^+^ Treg during the first and second wave of COVID-19. Dots show individual measurements of groups: HD (black), MD patients (blue), and SD patients (red), and black lines show the mean frequency of each population +/- SD **(B, C)**. In **(B, C)** one-way analysis of variance (ANOVA) followed by the Bonferroni multiple comparisons test or the Kruskal-Wallis test followed by Dunn’s multiple comparisons test were used when the measured variable had or had not met the normality assumptions, respectively.

Different T-cell subsets have distinct roles in both controlling viral infections and promoting tissue repair (33), including in SARS-CoV-2 infection (8, 34). Even though COVID-19 patients did not show differences in the frequency of total CD4^+^ T cells compared to HD, we wondered if deeper analysis of CD4^+^ T cell subsets might identify critical components of the immune response triggered by SARS-CoV-2 infection. Thus, we focused on CD4^+^ T cell subsets from COVID-19 patients recruited during the first and second waves of the infection. By multiparameter flow cytometry, we evaluated in CD3^+^CD4^+^ T cells identified by the expression of Foxp3 (Tconv: Foxp3^-^ and Treg: Foxp3^+^), the expression of molecules related to T cells function as well as activation/exhaustion. At a first sight, the radar plots depicted in **Figure 2A**, showed that, during the first and second wave of SARS-CoV-2 infection, cells expressing molecules associated with cytotoxicity such as Granzyme B (GZMB) and CD107a were more frequent in both Tconv and Treg populations from COVID-19 patients, as also were molecules linked to exhaustion such as CD39 and PD-1 (35), than in those from HD (**Figure 2A**).

Statistical analysis of the frequency of these surface marker-expressing Tconv cells from individual patients confirmed our first observation. Indeed, during the first wave, the frequency of GZMB, CD107a, CD39, and PD-1-expressing Tconv cells was significantly higher in MD and SD patients, but CD4+ Tconv cells showed no impairment in cytokine production. During the second wave, there were significant increases only in GZMB-expressing Tconv cells of moderate and severe patients. While MD and SD patients showed impairment of IL-2 production, only the former exhibited a decrease in the frequency of TNF-producing cells compared to HD. No changes were detected in the frequency of IFN-γ in MD or in SD patients (**Figure 2B**).

We evaluated the differentiation phenotype of Tconv lymphocytes using CCR7 and CD45RA, markers that discriminate naïve (N: CCR7^+^CD45RA^+^), central memory (CM: CCR7^+^CD45RA^-^), effector memory (EM: CCR7^-^ CD45RA^-^), and effector memory-expressing CD45RA (EMRA: CCR7^-^CD45RA^+^) Tconv cells. There were non-significant statistical differences in the frequency of these populations between HD, SD, and MD from the whole cohort of infected patients (**Figure S2A**), and in patients stratified according to the first and second waves (**Figure S2B**).

The study of the frequency of GZMB, CD107a, CD39, PD-1 and TIGIT-expressing Treg cells did not reveal any distinctive pattern in COVID-19 patients associated either with infection or with disease severity (**Figure 2C**). However, infected individuals from the first wave showed a significant increase in CD107a^+^ Treg cells and a tendency toward a higher frequency of GZMB^+^ cells compared to HD. In patients from the second wave, the percentage of GZMB^+^ Treg cells increased significantly with disease severity compared to HD, but there were no significant changes in CD107a expression. The evaluation of activation markers showed that, in the first wave, CD39^+^ Treg cells were significantly higher in SD COVID-19 patients, and there was a tendency toward a higher frequency of PD-1-expressing Treg cells compared to HD. In the second wave, MD and SD patients showed a higher frequency of GZMB-producing Treg cells, but only the former exhibited a higher frequency of PD-1 than HD. No significant difference was observed in TIGIT-expressing Treg cells (**Figure 2C**).

### COVID-19 patients exhibited an increased frequency of dysfunctional CD8^lo^ T cells

**Figure 3:**
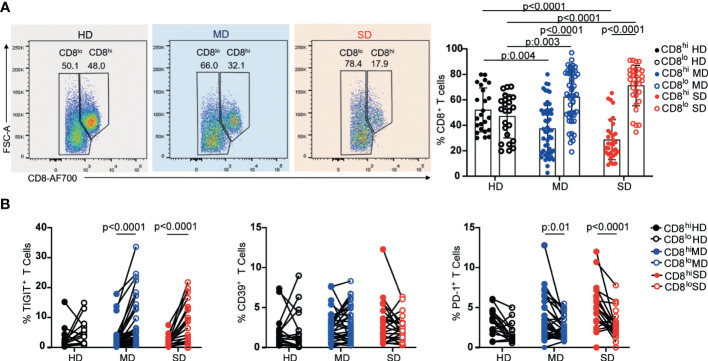
Frequency of CD8 high and low subpopulations and exhaustion markers from PBMC of HD and COVID-19 patients. Representative dot plots and graphs show frequencies of CD8^hi^ (filled circle) or CD8^lo^ T cells (open circle) from PBMCs of the entire cohort of HD and MD and SD COVID-19 patients. Dots show individual measurements. Data presented as mean +/- SD **(A)**. Frequencies of TIGIT^+^, CD39^+^, and PD-1^+^ within CD8^hi^ (filled circle) and CD8^lo^ T cells (open circle) **(B)**. Dots show individual measurements and lines indicate paired data. Two-way analysis of variance (ANOVA) followed by the Bonferroni multiple comparisons test were calculated in **(A, B)** for statistical analysis.

To analyze the CD8^+^ T cell response, we followed a similar approach to that used for the analysis of the CD4^+^ T cell response. Interestingly, when analyzing the expression of the co-receptor CD8 in a CD8 vs FSC-A dot plot, we observed that MD and SD COVID-19 patients from the entire cohort presented a significant increase in the frequency of CD8^lo^ T cells with respect to HD (**Figure 3A**). Indeed, whereas the percentages of CD8^hi^ and CD8^lo^ cells within the CD8^+^ population were similar in HD, there was a significant increase in the frequency of CD8^lo^ T cells in COVID-19 patients. Even though the differences in the frequency of CD8^lo^ T cells among MD and SD were not significant, we observed a tendency to increase in this population as the disease worsened (**Figure 3A**).

Given that CD8 co-receptor downregulation has been associated with activation (36) and chronic antigen stimulation (37, 38) we examined the frequency of the inhibitory receptors, TIGIT, CD39, and PD-1, associated with both activation and chronic antigen stimulation, in CD8^lo^ and CD8^hi^ T cell populations from the three groups under study. As can be seen in **Figure 3B**, we observed a significant increase in the frequency of TIGIT^+^ cells within CD8^lo^ T cell populations from MD and SD patients, while no differences were detected in the proportion of CD39^+^ cells between CD8^hi^ and CD8^lo^ subsets of any evaluated group. Regarding PD-1, compared to CD8^hi^ T cells, a lower percentage of PD-1^+^ T cells was seen within the CD8^lo^ subset in COVID-19 patients regardless of the severity of the disease.

**Figure 4:**
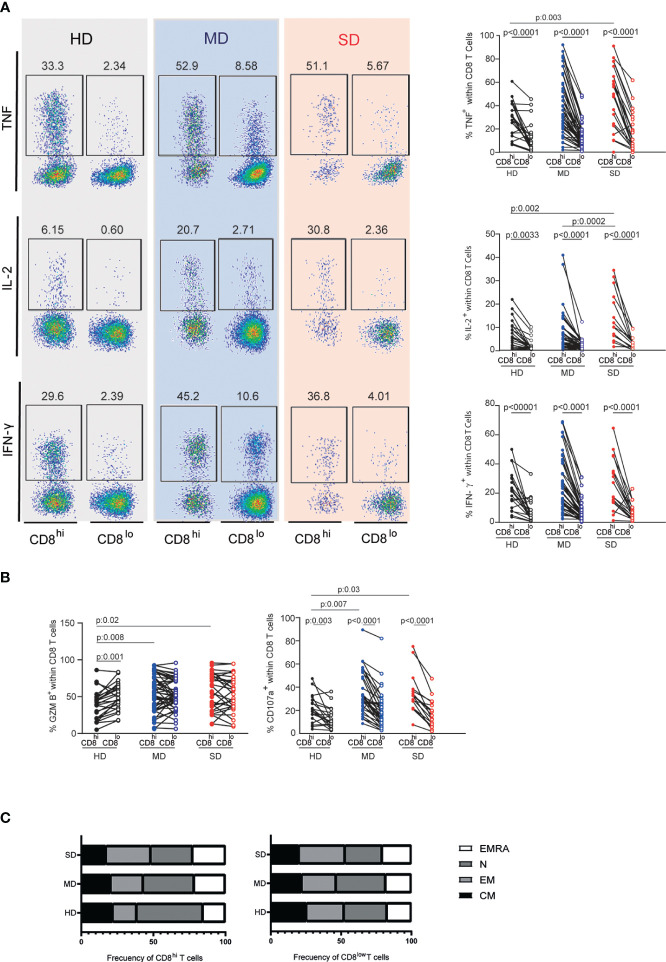
Assessment of cytokine-producing CD8^lo^ and CD8^hi^ T cells and their state of differentiation. Representative dot plots (left) and accumulated data (right) line graphs show frequencies of TNF, IL-2, and IFN-y-producing CD8^hi^ (filled circle) and CD8^low^ T cells (open circle) from PBMCs of the entire cohort of HD and MD and SD COVID-19 patients **(A)**. Frequency of GZMB and CD107a-producing CD8^hi^ or CD8^lo^ T cell populations of the entire cohort of HD and MD and SD COVID-19 patients **(B)**. Lines indicate paired data in **(A, B)** Differentiation profile of CD8^hi^ and CD8^lo^ subpopulations **(C)**. Two-way analysis of variance (ANOVA) followed by Bonferroni **(A, B)** or Tukey **(C)** multiple comparisons test was calculated for statistical analysis.

To study more deeply the functional capacity of CD8^lo^ and CD8^hi^ T cells subsets from the entire cohort of infected patients, we evaluated the frequencies of cytokine-producing cells and cytotoxicity-associated markers. **Figure 4A** shows that, after PMA/Ionomycin stimulation, CD8^lo^ T cell populations from HD as well as from SARS-CoV-2 infected patients (MD and SD) exhibited a lower frequency of the effector cytokine-producing cells, TNF, IL-2, and IFN-γ, than CD8^hi^ T cells. The significant differences in both populations from the groups evaluated were confirmed by statistical analysis (**Figure 4A**). We found that SD patients exhibited a higher frequency of CD8^hi^ T cells expressing IL-2 compared to MD patients. We did not find differences in the expression of other cytokines such as TNF and INFγ (**Figure 4A**).

Additionally, we did not find differences in the functional response (TNF, IL-2, INFγ production) of CD8^lo^ T cells from COVID-19 patients (MD or SD) and HD. The frequency of GZMB-producing CD8^+^ T cells from MD and SD patients was similar among both CD8^+^ T cell subsets. However, in HD, we found a higher frequency of GZMB^+^ T cells in the CD8^lo^ than in CD8^hi^ T cells (**Figure 4B**). Additionally, the frequency of CD107a-producing CD8^lo^ T cells was significantly lower than those in the CD8^hi^ population in the three groups studied (**Figure 4B**). Interestingly, SD patients showed higher frequencies of CD8^hi^ T cells expressing TNF, IL-2, GZMB, and CD107a than did HD (**Figures 4A, B**).

Together these results indicate that CD8^lo^ T cells from HD and infected patients exhibit similar dysfunctional behavior, suggesting that the dysfunction observed in the CD8^lo^ T cell population is inherent to this population itself, rather than a feature triggered by the infection. In addition, we observed that COVID-19 infection induced an activation state in CD8^hi^ T cells.

To understand the composition of CD8^+^ T cell subsets analyzed in terms of their differentiation profile, we evaluated the frequency of CM, EM, EMRA, and N cells in CD8^lo^ and CD8^hi^ T cell populations, using the combination of CCR7 and CD45 markers.

The study of CD8^hi^ T cells revealed that, compared to SD patients, HD were enriched in naïve T cells (HD vs SD p: 0.003). In concordance, we detected a significant increase in the frequencies of non-naïve T cells in SD patients (EM: HD vs SD p: 0.0009; CM: HD vs SD p: 0.003; EMRA: HD vs SD p: 0.009) (**Figure 4C**, left panel). In addition, the non-naïve compartment increased as the disease progressed (EM: MD vs SD p: 0.009; CM: MD vs SD p: 0.003). Thus, naïve T cells were fewer in SD than in MD patients (p: 0.03).

The distribution of CM, EM, EMRA, and N T cells was similar in the CD8^lo^ T cell subsets from HD and from infected patients (**Figure 4C**, right panel). In addition, the phenotypic and functional characteristics of CD8^lo^ and CD8^hi^ T cell subsets from SD and MD patients were similar in both waves (data not shown).

To better visualize the complexity of the data, **Figure S3** shows a heatmap ranking the frequency of the different lymphocyte populations from HD, MD, SD patients from both waves mentioned in this study. As expected, variables such as the frequency of CD3^+^ cells, CD19^+^ cells, CD8^lo^ T cells, GZMB-expressing Tconv and Treg cells and B cells, and PD-1^+^ expressing Tconv cells, highlighted in **Figure S3**, were modified mainly in SD patients.

### Clinical improvement of SARS-Cov-2 infected patients was associated with a higher CD8^hi^/CD8^lo^ ratio

**Figure 5:**
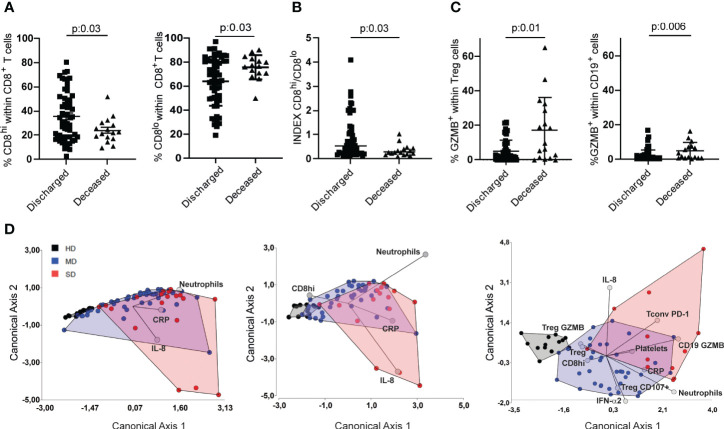
Determination of immune variables that enabled segregation of COVID-19 patients based on disease severity. Frequencies of CD8^hi^ and CD8^lo^ T cells of discharged and deceased COVID-19 patients **(A)** and their respective INDEX **(B)**. Frequencies of GZMB-producing Treg and CD19^+^ lymphocytes in discharged or deceased COVID patients **(C)**. Linear discriminant analysis (LDA) of parameters measured from the entire cohort. Patients were grouped in HD (gray), MD COVID-19 (blue), and SD COVID-19 (red), and graphs show their distribution in the canonical axes 1 and 2 **(D)**. P values were determined by the t-Test in A and by Mann-Whitney test **(B,C)**. Data presented as mean +/- SEM.

Several laboratory biomarkers are altered in patients with COVID-19, many of which are known to predict disease severity, hospitalization, and mortality, while others, such as metabolomic and proteomic analysis, have not yet been translated to clinical practice (39). As shown in **Figure 3A**, the frequency of CD8^hi^ T cells decreased as the severity of the disease increased, while the opposite was observed with CD8^lo^ T cell populations suggesting that this parameter may be considered a marker of disease severity. **Figure 5A** shows that, in both cohorts evaluated, discharged patients showed a higher frequency of CD8^hi^ T cells than deceased patients. Likewise, the former showed a decreased frequency of CD8^lo^ T cells.

Next, we decided to establish a CD8^hi^/CD8^lo^ index distinguishing clinically discharged patients from those with a fatal outcome. Indeed, patients with a clinical improvement exhibited a significantly higher CD8^hi^/CD8^lo^ ratio (**Figure 5B**). To investigate whether the CD8^hi^/CD8^lo^ index could be used as a marker for predicting disease severity, we compared the index between the entire cohort of COVID-19 patients with moderate and severe disease. Our findings revealed that SD patients exhibited a significantly lower CD8^hi^/CD8^lo^ index compared to MD COVID-19 patients (**Figure S4A**). We next analyzed the CD8^hi^/CD8^lo^ index according to the age and gender of the COVID-19 patients. We observed that, patients over 50 years of age exhibited CD8 ^hi^/CD8^lo^ ratios similar to those observed in patients under 50 years old. No differences were observed respect to gender (**Figures S4B, C**).

To add other variables that may have clinical significance, we included in the analysis the frequency of some lymphocyte populations together with markers that were modified mainly in SD patients (depicted in **Figure S3**). Thus, we analyzed the frequency of GZMB^+^ Treg and GZMB^+^ B cells in discharged vs deceased patients, and we found that patients discharged had the lowest frequencies of GZMB-expressing Treg and B cells (**Figure 5C**). Based on these findings, we evaluated whether the frequency of CD8^hi^ T cells might be a useful tool to incorporate into the panel of biomarkers that are commonly determined in clinical labs. To this end, we first performed a linear discriminant analysis (LDA) of the parameters measured in the three groups of donors of the entire cohort. Then, we calculated the apparent error rate (AER) when three commonly used laboratory biomarkers, such as neutrophil counts, C-reactive protein (CRP), and IL-8, were used to discriminate among the three groups (20). In this case, the AER was 29.1%, a value that is not significantly acceptable. When the frequency of CD8^hi^ T cells was included in the panel, the AER showed a slight reduction (AER=25.35%), **Figure 5D**, middle panel). Interestingly, when biochemical and inflammatory parameters were added as well as other immune cell populations selected based on our results, the AER reached levels as low as 13%, indicating the predictive capacity of this selected panel (**Figure 5D**, right panel). There was no strong correlation between any of the relevant quantitative variables included in the LDA (**Figure S5**).

Altogether, our data strongly support the addition of, at least, a CD8^hi^/CD8^lo^ index into the panel of biomarkers commonly used in clinical labs, since its determination could provide a better stratification with a clear impact on therapeutic management of the patients. Further studies would show whether changes in CD8 expression and alterations in the normal distribution of CD8^hi^ and CD8^lo^ T cell populations are also observed in other viral infection diseases.

## Discussion

In this study, we evaluated the phenotype and activation profile of the main peripheral blood lymphocyte populations of hospitalized patients with acute SARS-COV-2 infection, over the first (October 2020) and second wave (May 2021) of the pandemic in Córdoba in the central region of Argentina. To identify immunological parameters differentially expressed in circulating immune cells of COVID-19 patients with MD or SD, we evaluated the expression of activation/exhaustion surface markers, intracellular cytokines, and cytotoxicity-associated molecules in B lymphocytes, CD4^+^ and CD8^+^ T cells. Overall, our results agree with the findings reported for SARS-CoV-2 infection in different cohorts studied worldwide that COVID-19 patients experienced lymphopenia and disturbances in the normal frequency of circulating T and B cell subsets, with activated/exhausted and cytotoxic phenotypes (16, 40, 41). An enhance activation has been reported in all T cell subpopulation (CD8^+^, CD4^+^, T follicular helper cells, Treg) in patients progressing to severe COVID-19 (42), and severe disease and even fatal outcomes have been linked to excessive systemic proinflammatory response or cytokine storm. A hallmark of this dysregulated scenario is the lymphopenia with paradoxical CD8^+^ and CD4^+^ T cell hyperactivation or exhaustion, which mediate immunopathology or immunosuppression (41, 43, 44). Early studies during the pandemic showed that SARS-CoV-2 infection results in a broad reduction of innate immune cells as well as of T cell subsets (40) but, concomitantly, a relative expansion of B cells (16, 45). In agreement with these previous reports, we observed that COVID-19 patients exhibited a severe reduction of lymphocytes in peripheral blood compared to healthy individuals and presented significant changes in their normal distribution of circulating lymphocyte populations. COVID-19 patients progressing to severe disease had a reduction in the frequency of CD3^+^ T cells with a higher proportion of CD19^+^ cells within circulating immune cells. Therefore, it has been argued that lymphopenia in severe COVID-19 disease may arise due to T cell confinement in tissues or T cell apoptosis triggered by a pro-inflammatory cytokine environment (16, 40, 46). In the context of lymphopenia, a high frequency of circulating B cells has been described as an early event of SARS-CoV-2 infection (16, 47, 48). The expansion of B cell populations in severe COVID-19 patients has been associated with a maladapted immune response to the virus, reflecting polyreactivity, less viral control, and even contributing to tissue damage (46).

The deeper analysis of CD4^+^ T cells compartment revealed a decrease in the frequency of Treg cells in COVID-19 patients. A systematic review of Treg studies related to SARS-CoV-2 infection found a consistent pattern of decreased Treg levels in the peripheral blood of patients with severe cases of the disease (49). It is well known that Treg maintains immune homeostasis and prevents immunopathology in viral infections (50–52). In agreement, the reduced frequency of circulating Treg in patients with severe disease in our cohort matches the high levels of cytokines and chemokines we previously described in first wave hospitalized patients (20). The pro-inflammatory environment and the triad of high systemic levels of IP-10, IL-10, and IL-6 depicted in severe patients from the first COVID-19 wave from Argentina (20) have been reported in other cohorts, also occurring with a reduced frequency of circulating Treg (53). Mechanistically, Treg depletion may be a component of lymphopenia caused by apoptosis or extravasation into inflamed tissue, but it also may be a consequence of a direct inhibitory effect of proinflammatory cytokines, such as IL-6, on Treg differentiation (52, 54). Moreover, single-cell analysis in severe COVID-19 patients revealed that CD4^+^ T cells are hyperactivated, but Foxp3 expression is repressed (55).

We observed that, depending on the wave evaluated and the severity of the disease, CD4^+^ Tconv and Treg from hospitalized patients compared to HD displayed an activation/exhaustion pattern, determined by high expression of PD-1 and CD39, along with cytotoxic features. Similarly, functional impairment in CD4^+^ and CD8^+^ T cells has been frequently described in SARS-CoV-2 infection; however, it has not been clearly defined whether upregulation of PD-1 and other inhibitory markers means canonic T cells activation or exhaustion (41). An optimal T cell response to infections is contingent upon the proficient functioning of cytokine-producing cells. In COVID-19 patients, we did not observe severe impairment in cytokine production by Tconv in comparison with HD, suggesting that this population retain the ability to respond to stimuli. However, we detected high frequencies of PD-1^+^, CD39^+^, CD107a^+^, and GZMB^+^ CD4^+^ Tconv cells, particularly in patients during the first wave, suggesting that these T cells were activated and prone to be exhausted. In agreement with this, but in a different setting characterized by significant inflammation, it has been found that CD39^+^CD4^+^ tumor-infiltrating Tconv cells from patients with breast cancer display features of exhaustion while retaining the ability to produce effector cytokines (56). In agreement with our observation, cytotoxic CD4^+^ T cells producing GZMB, perforins, and CD107a, have been widely described in respiratory viral infections (43, 57) Particularly, in SARS-CoV-2 infection, Kaneko et al. (58) described significant expansion of cytotoxic CD4^+^ T cells in the lungs of patients with severe disease. In addition, high proinflammatory cytokine production in severely diseased patients is associated with persistent expansion of hyperactivated effector CD4^+^ and CD8^+^ T cells capable of secreting GZMB. Interestingly, this phenomenon has been related to excessive bystander activation of nonspecific T cells that might contribute to tissue damage (47). Remarkably, using single-cell transcriptomics and proteomics analysis, Georg et al. (59) demonstrated that severe COVID-19 is characterized by activated, highly cytotoxic, CD16-expressing T cells that promote microvascular endothelial cell injury through immune-complex-mediated degranulation. The cytotoxic T cell profile persisted for more than six months and was correlated with slower recovery rates (60). Similarly, our data suggest that the Tconv response in severe COVID-19 patients may be harmful, as these cells showed the expression of a cytotoxic profile, a feature associated with poor prognosis of the disease.

Our results also reveal that circulating GZMB-producing CD19^+^ cells contribute to predicting the outcome of clinical disease, as the frequency of these cells was significantly increased in deceased versus discharged patients (**Figure 5D** and **S3**). According to our data, in individuals infected with Epstein-Barr virus (EBV) or HIV there is an increased frequency of GZMB-producing peripheral B cells (61, 62). Kaltenmeier et al. (62) found high levels of GZMB-producing B cells in patients in early stages of HIV infection, along with IL-21-secreting T helper cells that barely expressed CD40L. In *in vitro* studies, IL-21 induced the differentiation of GZMB-producing B cells (61), empowering these cells with regulatory properties (63). Although we have not studied the expression of CD40L or IL-21 on Tconv in COVID patients, it is plausible that GZMB-producing B cells may play an immunoregulatory role and contribute to the immunopathogenesis of SARS-CoV-2, as they may be involved in T cell suppression. Further investigation into this mechanism could provide valuable insights.

Interestingly, in this study, we identified two subsets of circulating CD8^+^ T cells with low and high CD8 surface expression. We detected an expansion of T cells with low expression of CD8 in COVID-19 patients. Indeed, the CD8^hi^/CD8^lo^ index helped us to significantly improve the patient’s clinical stratification and disease outcome prediction. CD8^+^ T cell response patterns in COVID-19 patients are highly variable (41). Although robust CD8^+^ T cell activation is a response of many hospitalized COVID-19 patients, a considerable number of patients exhibited CD8^+^ T cell activation comparable with that in uninfected individuals (16). In agreement with our findings, studies have shown that human CD8^+^ T cells from peripheral blood contain a low CD8-expressing cytotoxic/effector subpopulation (64). Moreover, this CD8^lo^ T cell subset has been described to be expanded in viral infections triggered by HIV, EBV, and CMV (64, 65). The CD8^lo^ expansion may be a consequence of long-term, low-level response resulting from chronic and/or repeated exposure to antigens (64). Furthermore, a recent work has demonstrated that CD8^dim^ T cells are expanded in HIV-positive individuals with persistent Kaposi’s sarcoma and exhibit features of mitochondrial dysfunction (66). In another infection scenario, during response to *Listeria monocytogenes*, reduced CD8 expression was observed on T cell correlates with a decreased CD8^+^ T cell sensitivity for antigen (67). This CD8 downregulation is modulated by the antigen and type I interferon. These results suggest that effector CD8^+^ T cell differentiation involves a transient downregulation of antigen sensitivity via reduced CD8 expression.

CD8^lo^ or CD8^hi^ T cells were excluded to be CD3^+^/NKT cells co-expressing CD8. This was evaluated in HD as well as SD and MD COVID-19 patients when sample volume collected permitted for extra testing (data not shown). Interestingly, previous results from other group reported that severe patients had a significantly lower percentage of NKT cells respect to moderate COVID-19 patients and HD (68). Additionally, Kreutmair et al. (69) demonstrated a reduction in CD56^+^ T cells within the first week following COVID-19-related hospital admission. Then, based in this information we can conclude that the results obtained about CD8^hi^/^lo^ T cells are not biased by the frequency of NK or NKT cells.

Our data show that acute COVID-19 patients had a significantly higher proportion of the CD8^lo^ T cell subset within circulating CD8^+^ T cells than HD. This CD8^lo^ T cell subset seems to be inherently dysfunctional as they exhibited a profound impairment in the production of the effector cytokines, IL-2, IFN-γ, or TNF and upregulated TIGIT, a marker associated with exhaustion respect to CD8^hi^ T cells. Nevertheless, our data suggest that CD8^lo^ T cells maintain some cytotoxic properties since they produce GZMB. Likewise, Georg et al. (59) observed CD8^hi^ and CD8^lo^ T cell subsets within circulating CD8^+^ T cells from COVID-19 patients, although they did not focus their study on the phenotype or function of each subset. Interestingly, we also detected an impairment in cytokine production in CD8^lo^ T cells from HD, meaning that the dysfunction is inherent to CD8^lo^ T cell populations rather than to COVID-19 infection.

In contrast to the CD8^lo^ subpopulation, our data show that CD8^hi^ T cells may represent an activated population that upregulates PD-1, with the ability to respond with high cytokine and GZMB production and to degranulate, based on CD107a expression. In agreement with our results, several reports show that CD8^+^ T cells in patients with SARS-CoV-2 infection who develop severe disease are a heterogeneous population with hyperactivated CD38^+^HLA-DR^+^KI67^+^CD39^+^PD-1^+^, concomitantly with functionally exhausted NKG2A^+^CTLA-4^+^TIGIT^+^TIM-3^+^ phenotypes (16, 43, 53, 70). We also observed that infected patients harbored a higher proportion of effector memory cells (EM and EMRA) within the CD8^hi^ population and, in contrast, a higher frequency of T naïve phenotypes in the CD8^lo^ T cell subset than healthy controls. Considering that SARS-CoV-2 antigen-specific T cells have been identified within central or effector memory compartments (71), we speculate that the CD8^hi^ T cell subset may be able to better control a subsequent viral infection than CD8^lo^ T cells.

Our study thus demonstrates that the severity of SARS-CoV-2 infection in patients from both waves in Argentina correlates with changes in the distribution of subpopulations of CD8^+^ T cells in peripheral blood. Patients progressing to severe disease have a lower proportion of the activated CD8^hi^ T cell subpopulation (likely with better antiviral features), concomitantly with an increased frequency of a dysfunctional or exhausted CD8^lo^ T cell subset.

While our data present promising information regarding the impact of the CD8^hi^/CD8^lo^ index on patient stratification and subsequent therapeutic management, further research is needed to incorporate it as a biomarker in the clinical laboratory. Additional studies should be done to see if changes in CD8 expression and alteration of normal distribution of CD8^hi^ and CD8^lo^ T cells are also observed in other viral infections. In summary, our study offers valuable understanding of the cell-mediated immune response to SARS-CoV-2 infection in two pandemic outbreaks in Argentina that may be relevant when studying other viral infections that trigger systemic proinflammatory responses.

## Data availability statement

The raw data supporting the conclusions of this article will be made available by the authors, without undue reservation.

## Ethics statement

This study received approval from the “Registro Provincial de Investigación en Salud (RePIS)” (Provincial Registry of Health Research), Córdoba, Argentina under number 4039. The studies were conducted in accordance with the local legislation and institutional requirements. The participants provided their written informed consent to participate in this study.

## Author contributions

LIO, CM, JD, MB performed the experiments, analyzed data, and prepared the figures. EEB, SB, JQ, LA, FM, CO performed the experiments, analyzed data. SS-R analyzed data. NP and PI collaborated with the methodology. MCAV, BM, EVAR, LF, MCRG, CCS, LC conceived the project and collaborated with the general development of the study. ImmunoCovidCBA group collaborated with the project. MB, CA, DE, AK, and JC performed the recruitment and clinical evaluation of the patients. MM and CCM conceived the project, participated in the development of the study and discussion of results. CLM, CS, AG and LCH conceived the project, designed, supervised, provided overall direction for the study, and wrote the manuscript. All authors contributed to the article and approved the submitted version.

## Group members of ImmunoCovid-CBA

Fabio Cerban, Pablo Iribarren, Daniela Soledad Arroyo, and Gabriel Moron.

## Supplementary material

Supplementary Figure 1 Gate strategies of lymphocyte subpopulations from PBMCs of HD. **(A)** Gate strategies of effector molecules, CD39 and PD-1 expressing Tconv cells from COVID patients **(B)**. Gate strategies of effector molecules, CD39, PD-1 and TIGIT expressing Treg cells from COVID patients.

Supplementary Figure 2 Differentiation profile of CD4^+^ Tconv cells from PBMCs of the entire cohort of HD and MD and SD COVID-19 patients **(A)** or from patients from first and second wave analyzed separately **(B)**.

Supplementary Figure 3 Heat map of the frequencies of the different immunological variables tested from PBMCs of the entire cohort of MD and SD COVID-19 patients and HD. The numbers in each rectangle represent the media value of the variables. Arrows indicate the immunological variables included in the LDA (**Figure 5**).

Supplementary Figure 4 Index CD8^hi^/CD8^lo^ T cell populations from the entire cohort of MD and SD COVID-19 Patients **(A)** the entire cohort of MD and SD COVID-19 Patients analysed according age **(B)** the entire cohort of MD and SD COVID-19 Patients analysed according gender **(C)**. Dots show individual measurements. P value was determined by the Mann-Whitney test.

Supplementary Figure 5 Pairwise linear correlations across relevant quantitative variables derived from our cohort of COVID-19 patients. The distribution of each variable is displayed as a histogram. The scatter chart with the trendline (left) and Spearman’s rank correlation coefficient (right) for each comparison are depicted. The strength of correlation was defined as weak (0.00-0.39), moderate (0.40-0.59), or strong (0.60-1.00). Each correlation was considered statistically significant with a p-value less than 0.05.
