# Supplementary material for: COVID-19 patients display changes in lymphocyte subsets with a higher frequency of dysfunctional CD8lo T cells associated with disease severity
Source: Front Immunol. 2023 Sep 21;14:1223730. doi: 10.3389/fimmu.2023.1223730 (PMC10552777; doi:10.3389/fimmu.2023.1223730)
Supplement: Supplementary file 1 [file Table_1.docx]

| TABLE 1: Demographic and clinical characteristic of hospitalized COVID-19 patients | | | | | | |  |
| --- | --- | --- | --- | --- | --- | --- | --- |
|  |  |  |  |  |  |  |  |
|  |  | **First Wave** | |  | **Second wave** | |  |
|  |  | **Moderate** | **Severe** |  | **Moderate** | **Severe** |  |
| Patients (n) | | 20 | 10 |  | 32 | 23 |  |
|  |  |  |  |  |  |  |  |
| Age (years) | | 57.1 ± 13.2 | 56.7 ± 18.3 |  | 52.7 ± 12.3 | 58.4 ± 10.8 |  |
| Range (min-max) | | (30 - 80) | (24 - 89) |  | (31 - 76) | (42 - 78) |  |
|  |  |  |  |  |  |  |  |
| Gender | Men (n, %) | 14 (70%) | 9 (90%) |  | 23 (72%) | 17 (74%) |  |
|  | Female (n, %) | 6 (30%) | 1 (10%) |  | 9 (28%) | 6 (26%) |  |
|  |  |  |  |  |  |  |  |
| Age | Men (years) | 56.5 ± 14.4 | 54.1 ± 17.4 |  | 53.4 ± 13.8 | 57.8 ± 9.8 |  |
|  | Female (years) | 59.0 ± 8.5 | 80.0 ± 0.0 |  | 50.8 ± 7.7 | 60.2 ± 14.1 |  |
|  |  |  |  |  |  |  |  |
| Mortality | Men (%) | 0 % | 44.5% |  | 2.3 % | 41.5 % |  |
|  | Female (%) | 0 % | 100% |  | 0% | 66.7 % |  |
|  |  |  |  |  |  |  |  |
| n= number; age expressed as Mean ± SD; mortality expressed as % of deceased patients by gender in each group. | | | | | | | |
