# Supplementary material for: COVID-19 patients display changes in lymphocyte subsets with a higher frequency of dysfunctional CD8lo T cells associated with disease severity
Source: Front Immunol. 2023 Sep 21;14:1223730. doi: 10.3389/fimmu.2023.1223730 (PMC10552777; doi:10.3389/fimmu.2023.1223730)
Supplement: Supplementary file 2 [file Table_2.pdf]

Antigen	Clon	Fluorochrome	Brand
CCR7	GO43H7	PerCP	Biolegend
CD107a	eBioH4A3	FITC	eBioscience
CD19	HIB19	BV605	Biolegend
CD3	UCHT1	BV650	Biolegend
CD39	eBioA1	Super Bright 436	eBioscience
CD4	RPA-T4	PE-Cy5	eBioscience
CD45	2D1	APC-Cy7	Biolegend
CD45RA	HI100	BV785	Biolegend
CD8	SK1	AF700	Biolegend
FOXP3	PCH101	PE-Cy5.5	eBioscience
Granzyme B	GB11	PE	eBioscience
IFNg	4S.B3	BV785	Biolegend
IL-2	MQ1-17H12	PE	eBioscience
PD-1	EH12.2H7	BV711	Biolegend
TIGIT	A15153G	PE-Cy7	Biolegend
TNF	MAb11	APC	eBioscience

Supplementary Table S2
