# Supplementary figures and images for: COVID-19 patients display changes in lymphocyte subsets with a higher frequency of dysfunctional CD8lo T cells associated with disease severity
Source: Front Immunol. 2023 Sep 21;14:1223730. doi: 10.3389/fimmu.2023.1223730 (PMC10552777; doi:10.3389/fimmu.2023.1223730)

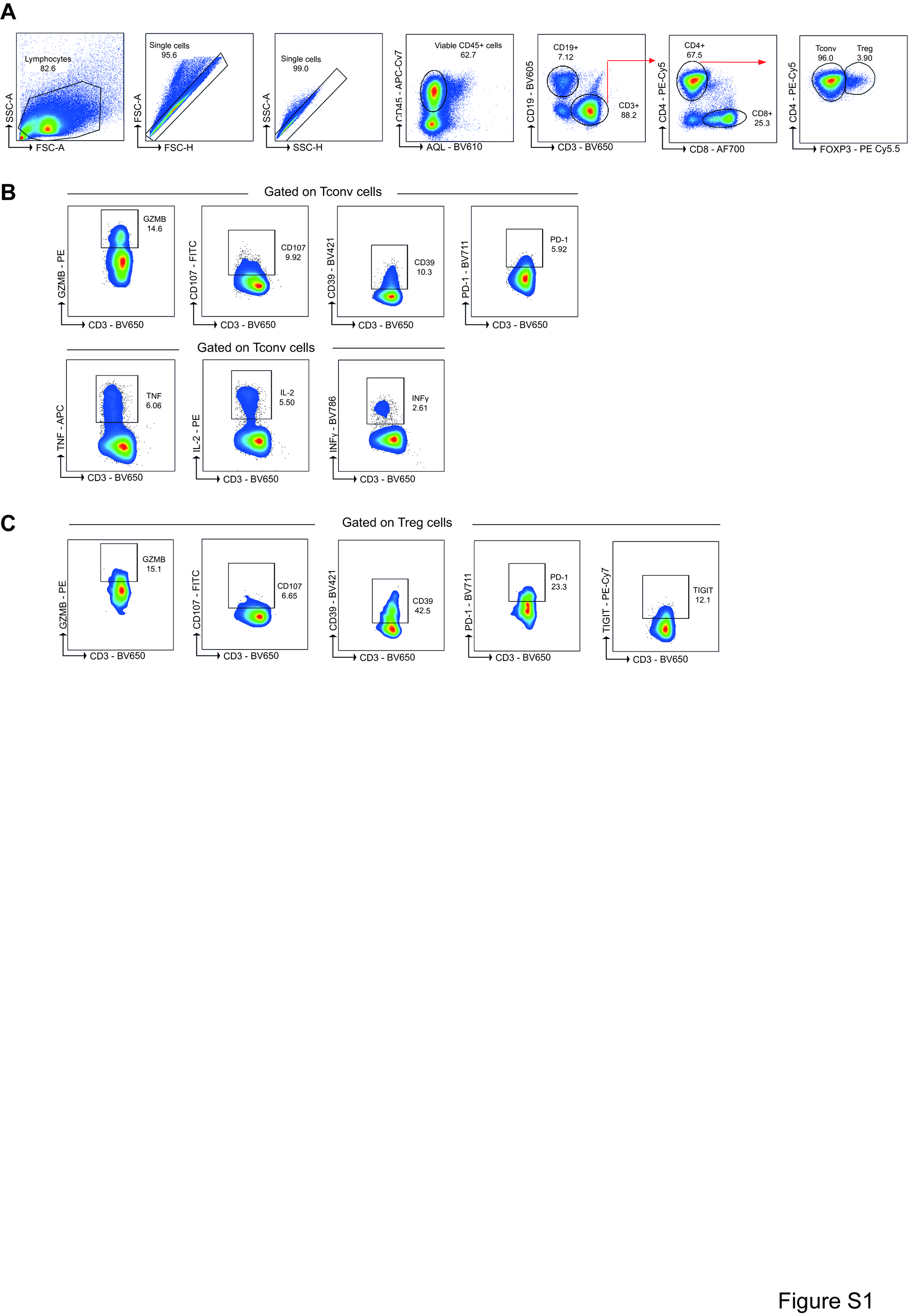

Supplement: Supplementary Figure 1 — Gate strategies of lymphocyte subpopulations from PBMCs of HD. (A) Gate strategies of effector molecules, CD39 and PD-1 expressing Tconv cells from COVID patients (B). Gate strategies of effector molecules, CD39, PD-1 and TIGIT expressing Treg cells from COVID patients. [file Image_1.tif]

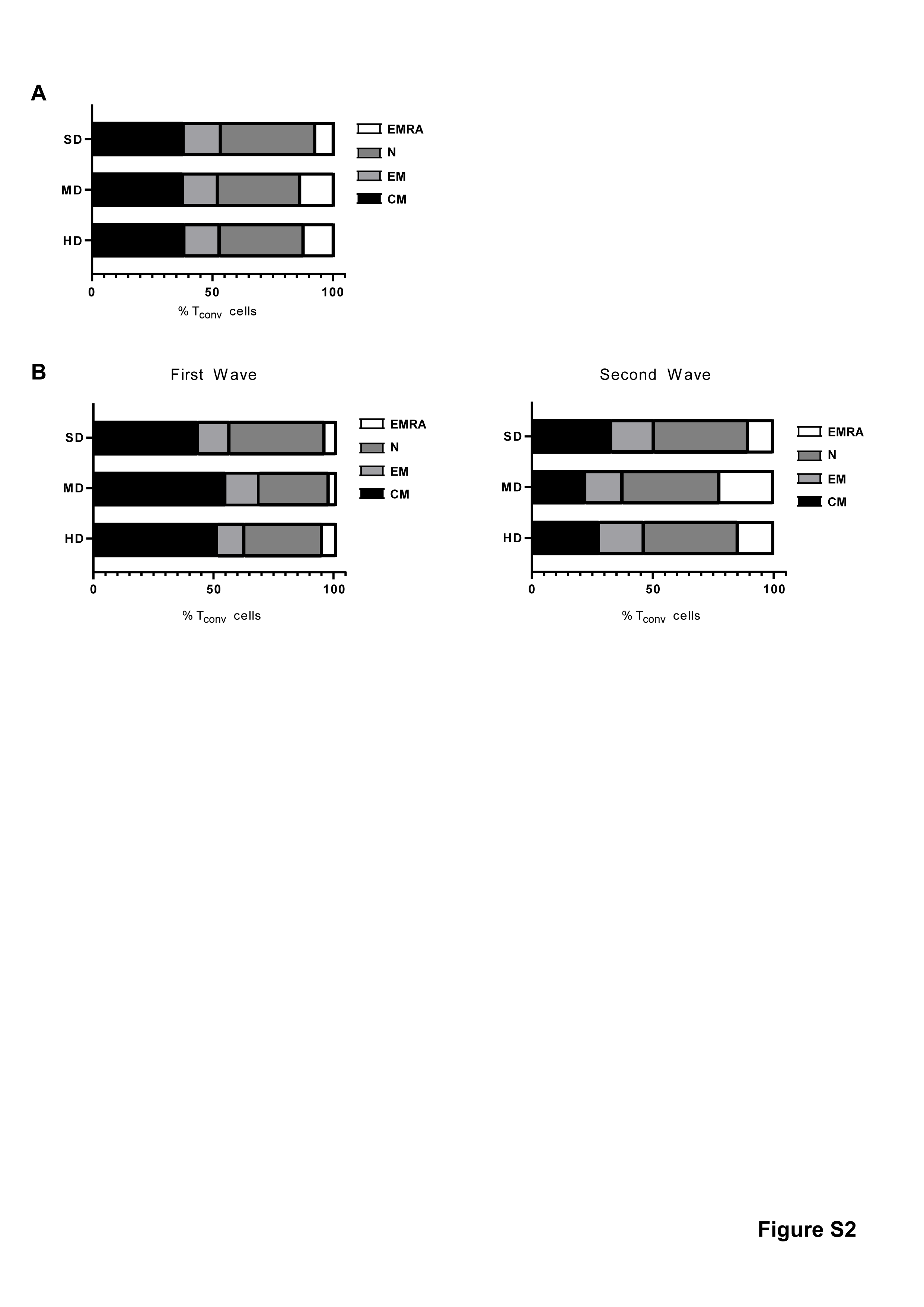

Supplement: Supplementary Figure 2 — Differentiation profile of CD4+ Tconv cells from PBMCs of the entire cohort of HD and MD and SD COVID-19 patients (A) or from patients from first and second wave analyzed separately (B). [file Image_2.tif]

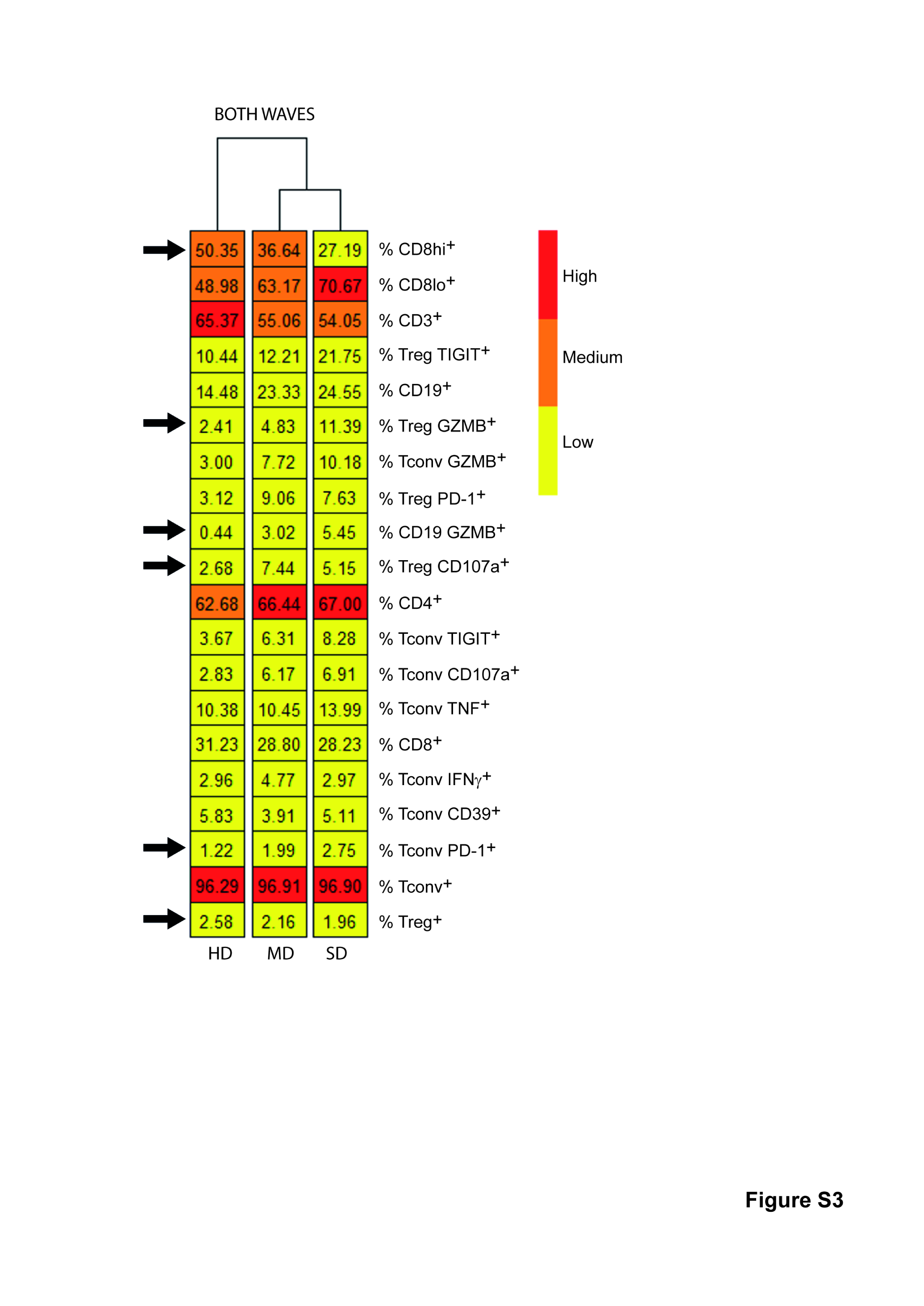

Supplement: Supplementary Figure 3 — Heat map of the frequencies of the different immunological variables tested from PBMCs of the entire cohort of MD and SD COVID-19 patients and HD. The numbers in each rectangle represent the media value of the variables. Arrows indicate the immunological variables included in the LDA ( Figure 5 ). [file Image_3.tif]

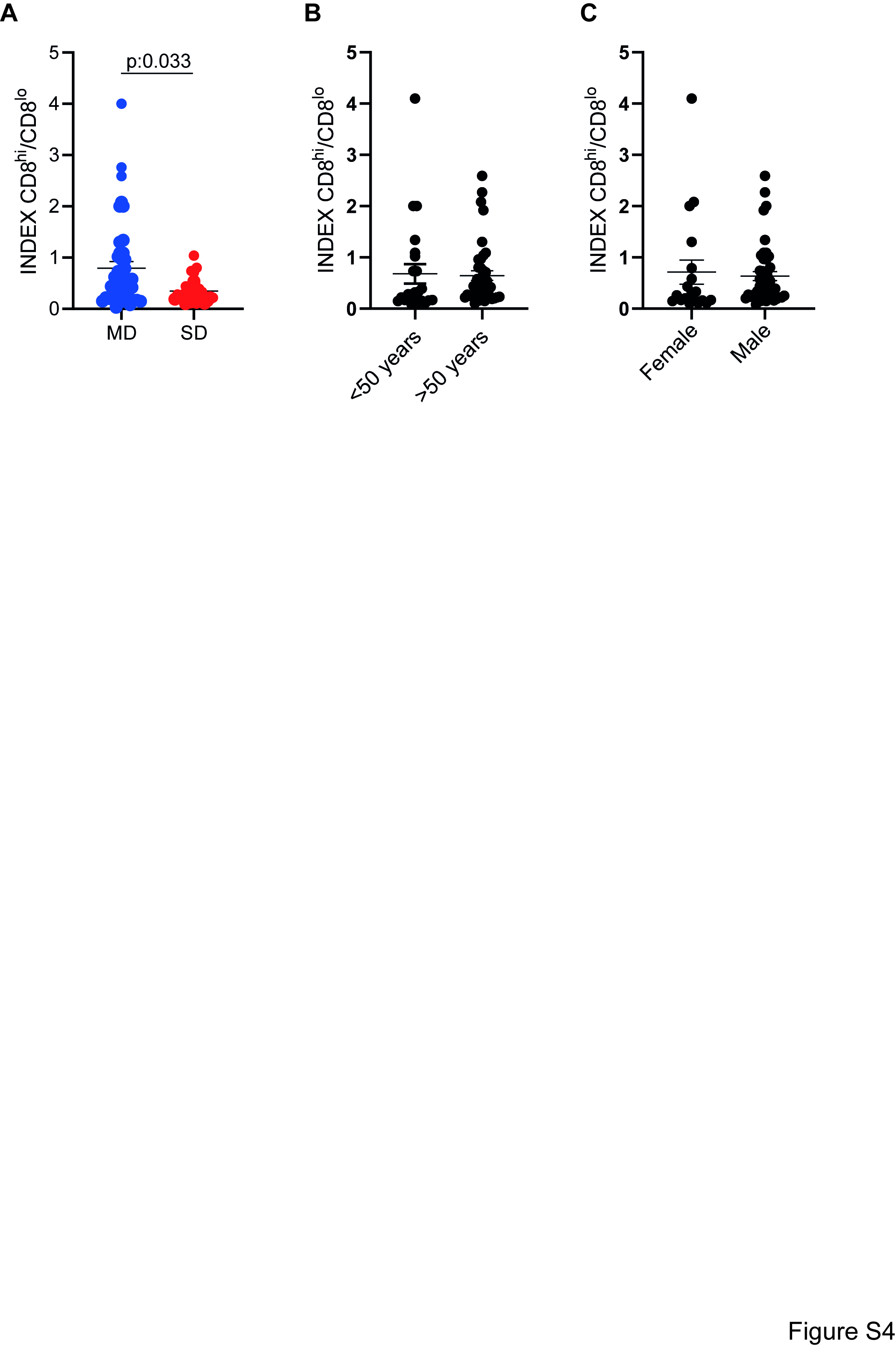

Supplement: Supplementary Figure 4 — Index CD8hi/CD8lo T cell populations from the entire cohort of MD and SD COVID-19 Patients (A) the entire cohort of MD and SD COVID-19 Patients analysed according age (B) the entire cohort of MD and SD COVID-19 Patients analysed according gender (C). Dots show individual measurements. P value was determined by the Mann-Whitney test. [file Image_4.tif]

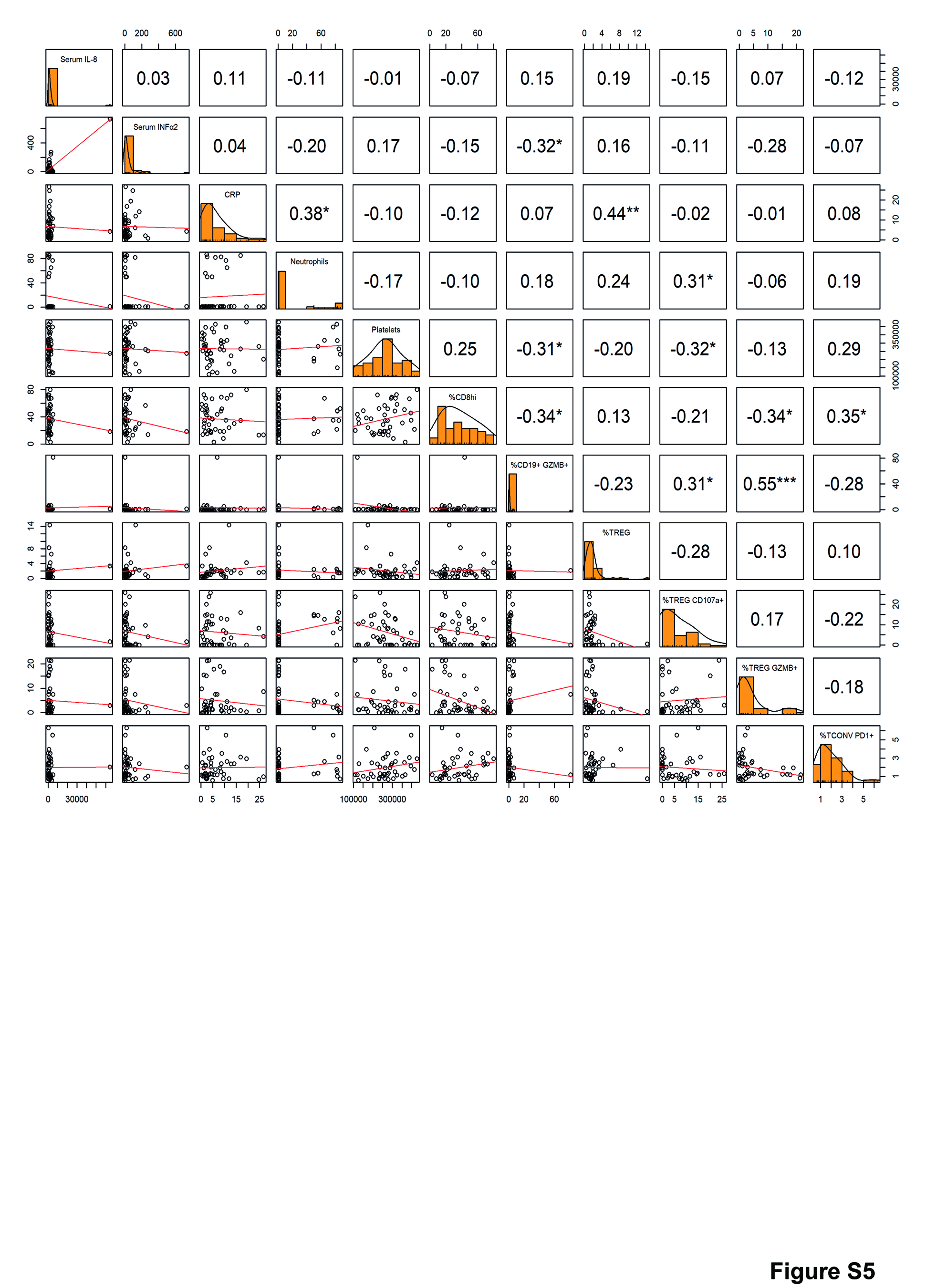

Supplement: Supplementary Figure 5 — Pairwise linear correlations across relevant quantitative variables derived from our cohort of COVID-19 patients. The distribution of each variable is displayed as a histogram. The scatter chart with the trendline (left) and Spearman’s rank correlation coefficient (right) for each comparison are depicted. The strength of correlation was defined as weak (0.00-0.39), moderate (0.40-0.59), or strong (0.60-1.00). Each correlation was considered statistically significant with a p-value less than 0.05. [file Image_5.tif]
